# Saliva and serum biomarkers in oral diseases: A case–control study

**DOI:** 10.1097/MD.0000000000041072

**Published:** 2024-12-27

**Authors:** Aliaa Al Shaar, Omar Hamadeh, Ayman Ali

**Affiliations:** a Department Of Oral Medicine, Faculty of Dental Medicine, Damascus University, Damascus, Syria; b Biomedical Photonics Laboratory, Department of Laser Physics And Technology, Higher Institute For Laser Research And Applications, Damascus University, Damascus, Syria; c Department of Internal Medicine, Faculty of Medicine, Tishreen University, Latakia, Syria.

**Keywords:** lichen planus, oral cancer, oral disease, recurrent aphthous stomatitis, saliva

## Abstract

Saliva stands out as an advantageous diagnostic fluid due to various reasons that make it a reliable and practical source for analysis. Its abundance facilitates easy and large-scale sample collection, enhancing its diagnostic utility. The simplicity of collecting and storing saliva samples, combined with its accessibility and cost-effectiveness compared to other body fluids, makes it an attractive choice for diagnostic purposes. This study aims to investigate and compare variations in salivary biomarkers with their serum counterparts in specific oral diseases such as recurrent aphthous stomatitis (RAS), lichen planus (LP), and oral cancer (OC). A case-control study was conducted in 55 individuals, including 15 patients with RAS, 13 patients with LP, 12 patients with OC, and a 15 individuals in the control group. Saliva and serum samples were collected after a specified fasting period, and various biomarker analyses immunoglobulin A (IgA), cortisol, lactate dehydrogenase (LDH) were conducted using laboratory techniques. In this study, an independent samples *t*-test was employed to investigate the significant differences between the experimental groups and the control group. The results indicated that there was a statistically significant difference in the salivary IgA levels, salivary cortisol levels, and salivary LDH levels (*P* = .000) (*P* = .016) (*P* = .001), respectively, as compared to the control group. However, no significant differences were observed in serum cortisol levels (*P* = .363) and serum IgA levels (*P* = .101) when compared to the control group. Interestingly, significant differences materialized when evaluating serum LDH levels (*P* = .048) in the OC group as compared to the control group. Overall, this research contributes to a deeper understanding of the utility of salivary biomarkers in diagnosing oral diseases and may pave the way for improved diagnostic and treatment strategies in the field of oral health.

## 1. Introduction

Saliva is a vital body fluid that plays a crucial role in maintaining oral cavity homeostasis. Composed of approximately 99% water and <1% protein content, saliva consists of a complex mixture of various macromolecules and bioactive components. Over 2000 proteins and peptides have been identified in saliva, contributing to its diverse biological functions within the oral cavity. Additionally, low-molecular-weight molecules such as DNA, RNA, metabolic byproducts, and microbial flora are also present in saliva. These molecules and components exist in specific natural proportions in both serum and saliva, performing their intended functions. Alterations in the relative levels of these components can serve as potential indicators that can be investigated and measured to detect various diseases, or to assess a patient’s response to treatment.^[1]^

Saliva serves as a multifunctional fluid with primary roles in protecting the oral mucosa and aiding in the digestive process. Its composition and properties enable it to fulfill several essential functions within the oral cavity. Saliva acts as a buffer, maintaining a moderate pH, facilitating moisturization, aiding in swallowing and taste perception, and providing protection to oral mucosal surfaces against chemical agents. Furthermore, saliva plays a crucial role in speech production, contributing to articulation and vocalization.^[2]^

Salivary fluid utilizes 2 primary mechanisms to combat infections. The first mechanism involves the presence of antibacterial and antifungal agents, such as salivary peroxidase, histatin, and lysozyme. These agents exhibit potent antimicrobial activity, directly targeting and inhibiting the growth of pathogenic microorganisms present in the oral cavity.^[3]^

The second mechanism involves specific immune factors present in saliva, namely immunoglobulins, including IgA, IgM, and IgG. These immunoglobulins play a critical role in the immune defense system, being responsible for phagocytosis. They recognize and bind to foreign antigens, facilitating their elimination through phagocytic processes. This immune response within saliva further enhances its ability to protect against infections.^[3]^

The composition of saliva, with its antimicrobial agents, immune factors, and antioxidants, allows it to serve as a reflection of the physiological state of the body. The presence of potential infections or imbalances can result in alterations in the concentration or activity of these salivary components. As a result, saliva can be considered functionally equivalent to serum in providing valuable insights into the overall health status of an individual.^[4]^

Saliva serves as an advantageous diagnostic fluid for a multitude of reasons. Firstly, its abundance readily allows for sample collection in large quantities, making it a reliable source for analysis. Moreover, the ease of collection and subsequent storage of saliva samples further enhances its diagnostic utility. The simplicity of saliva collection, in comparison to other bodily fluids, translates to convenience and cost-effectiveness, rendering it an appealing choice for diagnostic purposes. Additionally, patient acceptability is a key advantage, as saliva collection poses minimal discomfort and is well-accepted by individuals of various age groups, including children, and those with limited mobility. Furthermore, the noninvasive nature of saliva sampling improves patient compliance, reduces anxiety associated with invasive methods like serum sampling, and enables feasible collection for individuals with special needs or those afflicted by infectious diseases with high transmission risks. As an added benefit, the risk of transmitting high-risk infections, such as HIV, through saliva samples is notably low, lending further credence to the use of saliva as a preferred diagnostic fluid. Overall, the favorable attributes of saliva make it an optimal choice for diagnostic purposes, boasting patient acceptability, accessibility, and safety.^[5]^

The focus of our investigation lies within several oral diseases, namely recurrent oral stomatitis (RAS), lichen planus (LP), and oral cancer (OC). These conditions have been chosen due to their prevalence and significance within the field. By investigating the changes in salivary biomarkers and comparing them with their serum counterparts, we aim to deepen our understanding of these specific oral pathologies and potentially contribute to the development of improved diagnostic and treatment approaches.

Firstly, we will delve into the intricacies of recurrent aphthous stomatitis (RAS), a chronic inflammatory disorder affecting the oral mucosa. This condition, characterized by the recurrent appearance of singular or multiple painful ulcers with clear edges and a necrotic center, stands as the most prevalent and discomforting oral disease.^[6,7]^

Given the unknown etiology of RAS and its frequent association with immune disorders, numerous research endeavors have focused on the detection of immune-related factors present in saliva. Immunoglobulins, glycoprotein molecules found in serum and various body fluids including saliva, tears, and intestinal juice, exhibit the ability to interact with antigens that incited their synthesis. Their fundamental role entails antigen identification, binding, and subsequent activation of immune activities aimed at eradication. Notably, T-cells necessitate antigen recognition facilitated by antigen-presenting cells, while B-cells secrete antigen receptors in the form of immunoglobulins (Ig) present in biological fluids. Within the scope of our investigation, we will specifically concern ourselves with the immunoglobulin class IgA, which exists in 2 forms: monomeric and dimeric.^[8]^ Monomeric IgA can be found in serum, whereas the dimeric form primarily resides on mucosal surfaces, secreted by plasma cells in connective tissues. The secretory form of IgA, known as SigA, which is a glycoprotein-bound complex of 2 IgA molecules, is prominently present in secretory fluids and mucus. The principal function of SigA is pathogen immobilization and prevention of their adhesion to mucous membranes, thus aiding in the defense against invading pathogens.^[9]^

We shall now delve into the subject of LP, an autoimmune disease characterized by cell-mediated mucocutaneous pathology resulting from an anomalous response of T-cells to basal cells of the epidermis via a yet unidentified mechanism.^[10]^ While the most commonly affected sites of LP are the skin and oral mucosa, it can also manifest in the esophagus, genitals, conjunctiva, and skin appendages such as the scalp, hair, and nails. Lichen planus presents itself in 2 distinct forms: cutaneous and mucous.

The cutaneous form of LP is typified by pruritic, flat, polygonal, purple lesions that may take on a papular or plaque-like pattern. Frequently, white papules coalesce to form a characteristic network of lines known as Wickham’s network. Predilection for this cutaneous injury is observed in areas of skin folds, particularly the wrists and feet. Conversely, the mucous form of LP exhibits diverse clinical presentations encompassing plaque, papular, reticular, atrophic, erosive, and bullous forms. Multiple clinical forms may simultaneously affect a given patient. The posterior cheek mucosa represents the most frequently afflicted oral area.^[11]^

The development of LP lesions ensues from T lymphocyte-mediated attack on epithelial cells, triggered by various stimuli, including medications, psychological factors, and genetic predisposition. Psychological stress and its impact on immune functioning have been implicated in a subset of studies, attributing the etiology of LP to a range of psychological and physical disorders. Prolonged exposure to stress and psychological pressure can lead to alterations or amplifications in the production of various cytokines, such as IL-2, IL-12, IL-4, IL-5, IL-6, IL-10, IL-13, TNF-α, and IFN-γ. Consequently, the inflammatory process is initiated, resulting in disruption of immune regulation.^[12]^ Additionally, the neuroendocrine immune system modulates the activity of CD8 + cytotoxic immune cells, thereby augmenting catecholamine levels. This elevation induces programmed cell death of epithelial cells, a hallmark feature of the pathogenic mechanism of LP. Subsequently, increased catecholamine secretion leads to elevated cortisol levels, an essential downstream product for hypothalamic-pituitary axis activation in both serum and saliva.^[13,14]^

Finally, an important topic of discussion is OC. OC is classified as a malignant neoplasm originating from spiny cells and exhibits varying degrees of epidermal differentiation. It demonstrates a propensity for lymphatic, osseous, cerebral, and hepatic colonization. The most commonly affected areas within the oral cavity include the tongue, pharynx, lip, floor of the oral cavity, gingiva, hard palate, and vestibular mucosa. The development of OC, akin to other malignancies, arises from the accumulation of multiple mutations in cellular genes, often attributed to prolonged exposure to carcinogenic agents. These mutations lead to a transformation of the healthy oral mucosal lining, initiating the development of precancerous lesions that progress into invasive and metastatic cancerous growths.^[15]^

Detecting OC in its early stages poses significant challenges due to the limited ability of specialists to identify precancerous lesions and the insufficient awareness and compliance of the general population regarding regular OC screenings. As a response to this diagnostic need, various methods, including liquid biopsy, have been developed. The National Cancer Institute defines liquid biopsy as a diagnostic test performed on serum samples to detect circulating tumor cells released from the primary tumor into the serum stream. However, the utilization of liquid biopsy extends beyond serum specimens to include other bodily fluids such as saliva, urine, sweat, bile, and cerebrospinal fluid, owing to their inherent genetic information and biomarkers.^[16]^

Saliva, in particular, holds significant promise in the diagnosis of OC due to its direct contact with precancerous lesions and the presence of various biomarkers such as cytokines (e.g., IL-6, IL-8), immunoglobulins, insulin growth factor, and cancer antigens (e.g., CA-125, CA-19). Additionally, saliva contains the enzyme lactate dehydrogenase (LDH), which plays a pivotal role in anaerobic glycolysis, catalyzing the reversible reduction of pyruvate to lactate. LDH is ubiquitously present in the cytoplasm of all cells and can be detected in the extracellular space upon cell death. Research suggests that salivary LDH primarily originates from oral epithelial cells. Elevated levels of LDH have been observed in the context of various oral mucosal pathologies, including OC and precancerous lesions like oral leukoplakia, oral LP, and oral submucosal fibrosis. This phenomenon may be attributed to the Warburg effect, a metabolic alteration characterized by an increased reliance on anaerobic glycolysis by cancer cells to fulfill their energy requirements. The Warburg effect leads to heightened lactate production and subsequently raised LDH levels.^[17]^

## 2. Material and methods

### 2.1. Study design

A case-control study was conducted in the Al-Assad University Hospital, and the Department of Oral Medicine, Faculty of Dentistry, Damascus University. The study was carried out between September 2019 and April 2022. Ethical approval for the study was obtained from the Ethics Committee at Damascus University under approval number DN-241023-8-H7. And this study was registered in the ISRCTN with the number 16047134.

### 2.2. Inclusion and exclusion criteria

Participants aged 18 years and above of both genders, with a clinical and histopathological examination, who did not have any systemic diseases such as hypertension, cardiovascular disorders, liver or kidney diseases, diabetes, or any other oral mucosal lesions that could affect the composition and secretion of saliva, were included. None of the participants were on any medication regimen. This study comprised 55 individuals, allocated into 4 groups as follows: 13 patients diagnosed with LP, 15 individuals confirmed to have RAS, 12 patients with OC, and 15 individuals representing the control group. The allocation of participants into these groups was determined using the G*Power 3.1.9.2 program with a significance level set at 0.05, confidence level at 0.95, and a size effect of RAS group at 1.25, a size effect of LP group at 1.35 and a size effect of OC group at 1.4. (Franz Faul, Universität Kiel, Germany).

### 2.3. Method of collection of data

Informed written consent was obtained from all participants after providing them with comprehensive information about the purpose and details of the study. Patients were included in the study after being diagnosed with LP, RAS, and OC based on both oral and histological examinations. Demographic data, including age, gender, and smoking status, smoking duration, presence of lymphadenopathy, number of oral lesions for the OC group, and lesion number, lesion duration and lesion location for the RAS group, as well as the lesion pattern, lesion location and presence of mucosal lesion with skin lesions within the LP group, were recorded. Serum and unstimulated saliva samples were collected from both the study and control groups between 8:00 am and 9:00 am after a 2-hour fasting period for saliva collection (except for the LP group where patients were asked to fast for 8 hours before sample collection to assess morning cortisol levels in both serum and saliva), and refrained from drinking, eating, or smoking during the fasting period.

### 2.4. Saliva and serum sample collection

Saliva samples were collected in 3 stages within a ten-minute interval after rinsing the mouth with distilled water, and the accumulated unstimulated saliva was spat into individual custom 2 ml containers. These samples were then transferred to a laboratory centrifuge and centrifuged for 3 minutes at 3000 rpm to remove salivary proteins and obtain clear saliva in the supernatant for analysis using a Hitachi 911 automated clinical chemistry analyzer to analyze IgA and LDH biomarkers.^[18]^ The enzyme-linked immunosorbent assay (ELISA) test was performed to measure the optical absorbance at a wavelength of 450 nm, with a standard curve allowing for the determination of cortisol concentrations ranging from 2.7 to 270.7 nm within the saliva samples and from 12.3 to 1656 nm to analyze cortisol levels.^[19]^ Similar procedures were followed for the examination of serum samples.

### 2.5. Statistical analysis

Data were tabulated and analyzed using SPSS software (SPSS Version 20, IBM SPSS Inc., Chicago). Statistical tests such as *t*-test, ANOVA were used to test the research hypotheses. Results were considered significant if *P* value < .05. and the results were presented in appropriate tables.

## 3. Results

**Figure 1.**
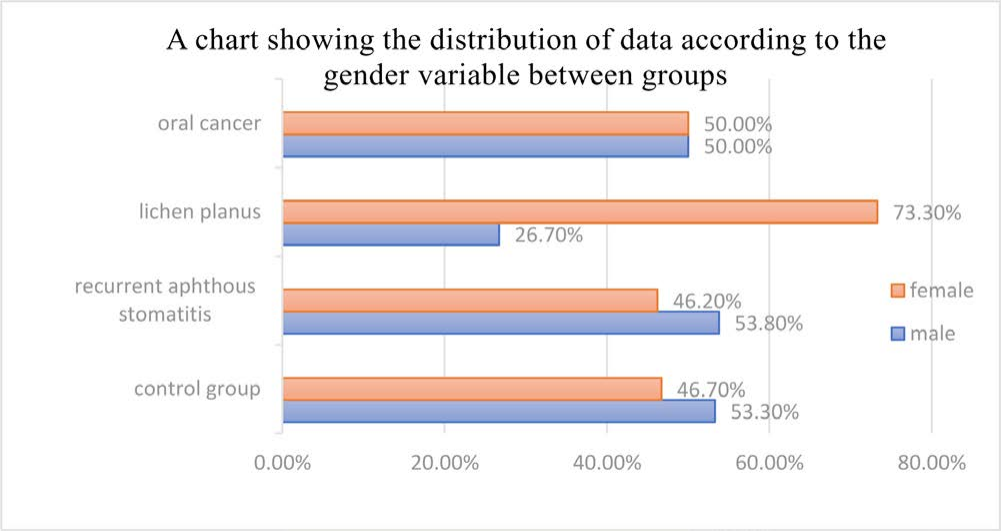
Distribution of data according to the gender variable between groups.

A total of 55 individuals participated in this study. There were 13 patients with RAS, comprising 7 males (53.8%) and 6 females (46.2%). The age range of these patients spanned from 19 to 26 years, with a mean age of 22.23 years with standard deviation 2.09. Of the total sample, 12 patients were diagnosed with OC. This subgroup had an equal distribution between males and females, accounting for 50% each. The age range within this subgroup was 33 to 80 years, with an average age of 57.67 years with standard deviation 13.98. Additionally, the study included 15 individuals diagnosed with LP, with 7 males (26.7%) and 8 females (73.3%). The age range of this subgroup was 26 to 71 years, with a mean age of 46.13 years with standard deviation 14.08. Lastly, the control group comprised 15 individuals, with 8 males (53.3%) and 7 females (46.7%). The age range within the control group was 19 to 29, with an average age of 24.4 years with standard deviation 2.95, as presented in Table 1 and Figure 1.

**Table 1:** Descriptive statistics of this study.

Groups	Number	Average age	Standard deviation	Male		Female	
				Number	Percentage	Number	Percentage
Control	15	24.4	2.95	8	53.3%	7	46.7%
RAS	13	22.23	2.09	7	53.8%	6	46.2%
OC	12	57.67	13.98	6	50%	6	50%
LP	15	46.13	14.08	7	26.7%	8	73.3%

### 3.1. Oral findings

**Figure 2.**
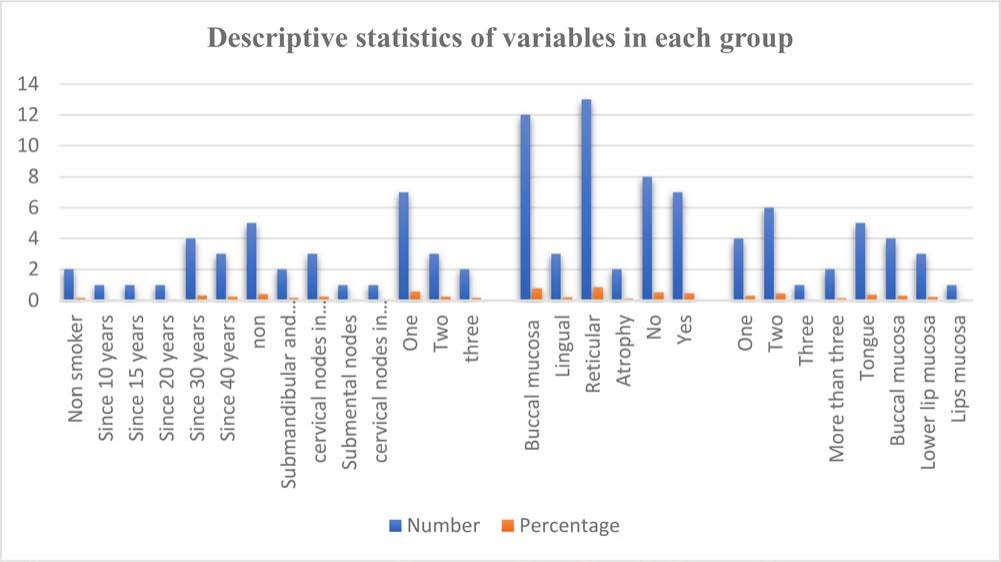
Descriptive statistics of variables in each group.

Within the OC group, a total of 10 individuals were identified as smokers, accounting for 83.3% of the group. The duration of smoking varied, ranging from 10 years (8.3%) to 40 years (25%). Lymphadenopathy was absent in the majority of cases (41.7%), while only 3 cases exhibited enlarged cervical lymph nodes on the same side as the lesion. In terms of the number of lesions, a single lesion was observed in 7 cases, while 2 lesions were recorded in 3 cases, and 3 lesions were present in 2 cases. In the LP group, the buccal mucosa was the most commonly affected site, accounting for 80% of the cases, whereas the tongue was affected in 20% of cases. Among the diagnosed cases, 13 were categorized as the reticular type, and 2 were classified as the atrophic type. Approximately 46.7% of the cases were accompanied by a concurrent skin lesion, while the remaining cases were localized to the mucous membrane. Among the recurrent oral stomatitis group, the highest percentage of ulcers documented was 2 ulcers (46.2%), while the smallest percentage constituted 3 ulcers (7.7%). The tongue was the most frequently affected site, observed in 5 cases (38.5%), while 4 cases were found on the buccal mucosa (30.8%). as presented in Figure 2.

**Figure 3.**
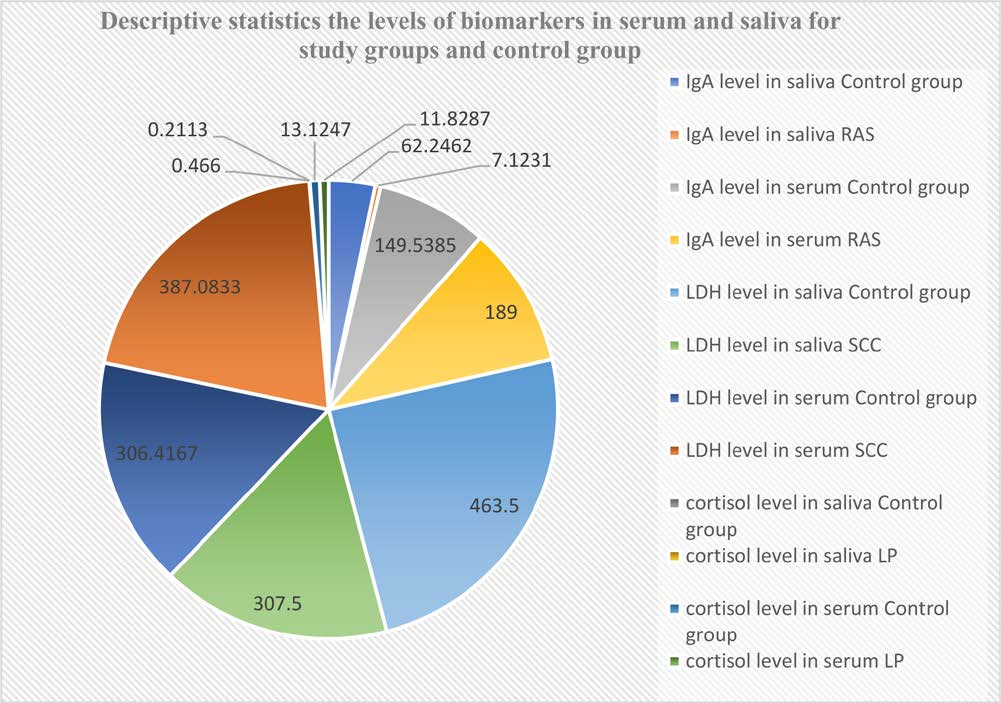
Descriptive statistics the levels of biomarkers in serum and saliva for study groups and control group.

The current study utilized an independent samples *t*-test to examine statistically significant differences between the experimental groups and the control group. Analysis revealed a significant difference in salivary IgA levels (*P* = .000) when comparing to the control group in RAS group. And significant difference was also observed in salivary cortisol levels (*P* = .016) when comparing to the control group in the LP group, whereas no statistically significant difference was observed in serum cortisol levels (*P* = .363) within the LP group compared to the control group. Similarly, there were no statistically significant difference in serum IgA levels (*P* = .101) within the RAS group compared to the control group. In contrast, significant differences emerged when evaluating both salivary LDH (*P* = .001) and serum LDH (*P* = .48) levels in the OC group when compared to the control group as presented in Table 2 and Figure 3.

**Table 2:** Descriptive analysis and study of the presence of a significant difference parameters in serum and saliva in study groups compered to control group.

Variables		Numbers	Mean	Standard deviation	*P* value	Significance test
IgA level in saliva	Control group	13	62.2462	5.89907	.000	There is a statistically significant difference
	RAS	13	7.1231	0.29530		
IgA level in serum	Control group	13	149.5385	13.67883	.101	There is no statistically significant difference
	RAS	13	189.0000	18.55657		
LDH level in saliva	Control group	12	463.5000	114.14703	.001	There is a statistically significant difference
	SCC	12	307.5000	69.51193		
LDH level in serum	Control group	12	306.4167	57.23390	.048	There is a statistically significant difference
	SCC	12	387.0833	120.75780		
Cortisol level in saliva	Control group	15	0.4660	0.36482	.016	There is a statistically significant difference
	LP	15	0.2113	0.12029		
Cortisol level in serum	Control group	15	13.1247	4.52975	.363	There is no statistically significant difference
	LP	15	11.8287	2.99296		

The present study employed the ANOVA test to elucidate potential differences in the variables across distinct groups. The result showed that there was no statistically significant difference between the number of lesions, smoking duration, Lymphadenopathy, and serum and saliva LDH levels among individuals in the OC group. Additionally, no substantial differences were found in the number of lesions, lesion types, associated skin lesions, and serum and saliva cortisol levels in the LP group. Furthermore, no statistically significant variations were observed in the number of lesions, lesion locations, and serum and salivary IgA levels in the RAS group, with *P* values > .05 for all parameters analyzed as presented in Table 3.

**Table 3:** Study of a significant difference between the levels of serum and saliva biomarkers and the research variables.

Variables			Sum of squares	Degree of freedom	Mean squares	*P* value	Statistically significant
*Study of a significant difference between the levels of LDH and the research variables*							
Number of lesions	The levels of LDH in the saliva	Between groups	6278.333	2	3139.167	.568	There is no statistically significant difference
		Within groups	46,872.667	9	5208.074		
		Total	53,151.000	11			
	The levels of LDH in the serum	Between groups	44,276.893	2	22,138.446	.234	There is no statistically significant difference
		Within groups	116,130.024	9	12,903.336		
		Total	160,406.917	11			
Smoking duration	The levels of LDH in the saliva	Between groups	20,267.583	5	4053.517	.621	There is no statistically significant difference
		Within groups	32,883.417	6	5480.569		
		Total	53,151.000	11			
	The levels of LDH in the serum	Between groups	89,498.917	5	17,899.783	.312	There is no statistically significant difference
		Within groups	70,908.000	6	11,818.000		
		Total	160,406.917	11			
Lymphadenopathy	The levels of LDH in the saliva	Between groups	11,881.533	4	2970.383	.735	There is no statistically significant difference
		Within groups	41,269.467	7	5895.638		
		Total	53,151.000	11			
	The levels of LDH in the serum	Between groups	72,573.050	4	18,143.263	.314	There is no statistically significant difference
		Within groups	87,833.867	7	12,547.695		
		Total	160,406.917	11			
*Study of a significant difference between the levels of cortisol and the research variables*							
Lesion location	The levels of cortisol in the saliva	Buccal mucosa	12	0.20750	.033710	.815	There is no statistically significant difference
		Lingual	3	0.22670	.092800		
	The levels of cortisol in the serum	Buccal mucosa	12	11.7492	.806470	.846	There is no statistically significant difference
		Lingual	3	12.1467	2.55532		
Lesion pattern	The levels of cortisol in the saliva	Reticular	13	0.20000	.031910	.372	There is no statistically significant difference
		Atrophy	2	0.2850	.125000		
	The levels of cortisol in the serum	Reticular	13	11.7415	.741880	.786	There is no statistically significant difference
		Atrophy	2	12.3950	4.40500		
With skin lesion	The levels of cortisol in the saliva	No	8	0.2275	.049920	.597	There is no statistically significant difference
		Yes	7	0.1929	.037330		
	The levels of cortisol in the serum	No	8	12.1025	.958210	.720	There is no statistically significant difference
		Yes	7	11.5157	1.31571		
*Study of a significant difference between the levels of IgA and the research variables*							
Lesion number	The levels of IgA in the saliva	Between groups	3.645	3	1.215	.399	There is no statistically significant difference
		Within groups	9.958	9	1.106		
		Total	13.603	12			
	The levels of IgA in the serum	Between groups	18,366.167	3	6122.056	.266	There is no statistically significant difference
		Within groups	35,351.833	9	3927.981		
		Total	53,718.000	12			
Lesion location	The levels of IgA in the saliva	Between groups	2.706	3	.902	.552	There is no statistically significant difference
		Within groups	10.897	9	1.211		
		Total	13.603	12			
	The levels of IgA in the serum	Between groups	9823.783	3	3274.594	.591	There is no statistically significant difference
		Within groups	43,894.217	9	4877.135		
		Total	53,718.000	12			

## 4. Discussion

The utilization of saliva as a diagnostic tool possesses several advantages when compared to serum tests and other diagnostic methods. This can be attributed to factors such as the ease of collection, safety, non-invasiveness, accuracy, and lack of vein damage that may occur during serum drawing. In this particular study, the focus was on cortisol, LDH, IgA as significant biomarkers, in order to compare its levels between serum and saliva in both the affected and control groups.

RAS, originally referred to as “aphthai” by Hippocrates, is characterized by small ulcers. IgA immunoglobulin plays a crucial role in safeguarding oral mucosal surfaces by inhibiting the adhesion of pathogens to mucous or tooth surfaces, serving as the primary defense against oral microbial agents. We observed a significant increase in salivary IgA levels in patients with RAS during the acute phase, which is consistent with previous studies conducted by Brozović et al,^[20]^ Sistig et al,^[21]^ Martinz et al,^[22]^ Saluja et al,^[23]^ Halboub et al.^[24]^ and Pakfetrat et al.^[25]^ This elevation in salivary IgA may be attributed to its innate immune response function in counteracting potential damage to mucosal epithelial cells by neutralizing antibodies and protecting mucosal surfaces. However, a single study reported no statistically significant difference in salivary IgA levels between the RAS and control groups. This discrepancy could be due to variations in disease stage during immunoglobulin titration, as well as variations in methodologies employed in immunoglobulin quantification.^[26]^

In our study, no significant difference in serum IgA levels was observed when compared to the control group. This finding aligns with a study by Al-Jojo et al, which reported no statistical difference in serum IgA levels between the study and control groups.^[27]^ Nonetheless, it is worth noting that IgA plays a crucial role in protecting mucous membrane surfaces, acting as a barrier against microbial adhesion. Any impairment to the immune system can result in increased susceptibility to infections and heightened incidence of autoimmune disorders. Compared to IgE and IgG in serum, IgA levels are comparatively lower. Consequently, changes in serum IgA levels are minimal, as corroborated by our study and in agreement with the findings of Halaboub et al.^[24]^ On the other hand, Brozović et al^[20]^ observed an increase in serum IgA levels during the acute phase of infection in their study sample when compared to the control group.

LP is a chronic T-cell-mediated inflammatory autoimmune disease that affects the oral cavity and skin, especially in women. The age of onset of OLP ranges between the third and sixth decades of life. Cortisol plays a crucial role in metabolism, vascular response, immune regulation, cognition, and biological regulation of behavior. Measuring cortisol levels can help analyze the activity of the Hypothalamic-Pituitary axis and assess the role of psychological factors in the occurrence of LP. Cortisol is also involved in various illnesses, including immunological disorders associated with inflammation. A significant difference was observed by comparing saliva cortisol between patients with LP and the control group, indicating an increase in the level of salivary cortisol among patients with LP. These findings align with previous studies, Koray et al,^[28]^ Ingafou et al^[29]^ and Humberto et al,^[11]^ which have also reported higher salivary cortisol levels among patients with LP when compared to the control group. Consequently, supporting the importance of employing saliva biomarkers in the diagnostic process. However, a comparative clinical investigation conducted by Pippi et al^[30]^ found that individuals with oral LP exhibited lower levels of salivary cortisol as compared to the control sample. This contradicting finding may be attributed to biological factors and variations in analytical techniques, which may have influenced hormone analyses, thereby leading to divergent results.

In addition, several studies, including those by Rödström et al,^[31]^ Girardi et al^[32]^ and Nosratzehi et al^[33]^ discovered no statistically significant difference in salivary cortisol levels between the group affected with LP and the control group.

Our study found no significant difference between serum cortisol in patients with LP and the control group. However, this disagrees with the findings of Muhasena et al,^[34]^ Jose et al^[35]^ and Chaitanya et al^[36]^ who reported higher serum cortisol levels in OLP patients compared to the control sample. This discrepancy may be attributed to various factors, including the method of analysis.

OC poses a significant global public health challenge due to its high incidence and mortality rates. Currently, the standard diagnostic approach involves an oral clinical examination followed by tissue biopsy. However, by the time most cases are diagnosed, they have already reached advanced stages. To address this issue, researchers have suggested salivary biomarkers as a noninvasive and relatively inexpensive diagnostic and screening tool for oral diseases, including OC. One such biomarker is lactate dehydrogenase (LDH), which has been proposed as a diagnostic and prognostic indicator for OC. In our study a significant difference in saliva and serum LDH was observed when compared to the control group. These findings can be explained by 2 proposed mechanisms from previous research. The first mechanism suggests that elevated LDH levels in saliva may be associated with the histological state of the oral mucosa, including ulceration, hypoxia, and fibrosis, which can lead to damage to oral epithelial cells. The second mechanism proposes that the increased LDH levels may be related to the “Warburg effect,” where cancer cells engage in anaerobic glycolysis, resulting in elevated lactate production. These mechanisms align with the findings of previous studies by Spitzer et al,^[37]^ Mantri et al,^[38]^ Shetty et al,^[39]^ Divyalakshmi et al^[40]^ and Kalali et al^[41]^ which also reported higher salivary LDH levels in the OC group compared to the control group and precancerous lesion group.

Additionally, previous research by Patel et al,^[42]^ D’Cruz et al^[43]^ and Lokesh et al^[44]^ suggests a correlation between tumor differentiation status and salivary LDH levels. These studies found that poorly differentiated cancer patients had significantly higher salivary LDH levels than those with well-differentiated tumors.

Consistent with prior investigations conducted by^[37,45–49]^ our study also observed an elevation in serum lactate dehydrogenase (LDH) levels in the examined group compared to the control group. This finding aligns with the established understanding that cancer cells engage in anaerobic glycolysis, a process that enables their rapid proliferation by generating the necessary energy. By corroborating these previous reports, our study supports the notion that increased serum LDH levels can serve as a relevant marker for the presence and progression of cancer.

The present study aims to investigate the diagnostic potential of saliva for oral diseases that cause painful symptoms that significantly impact patients’ quality of life. By analyzing various parameters in saliva and comparing them with their counterparts in blood, the study assesses the diagnostic ability of these biological fluids. The primary objective is to validate the reliable diagnostic value of saliva, which can serve as an alternative to blood samples. However, it is essential to acknowledge the study’s limitations, such as the use of a single parameter for each specific disease and a sample population sourced entirely from Syria, which could preclude generalizing the findings. Furthermore, the COVID-19 outbreak limited the sample size, thereby necessitating the need for future studies with more extensive sample sizes. Consequently, further research on liquid biopsy that utilizes saliva as a safe, accessible, and cost-efficient diagnostic tool for oral diseases and various diseases is imperative for generalizing the findings.

## 5. Conclusion

Saliva’s multifaceted role in oral health includes protecting the oral mucosa, aiding the digestive process, and possessing antibacterial, antifungal, immune, and antioxidant properties. Its diagnostic potential as a noninvasive and easily accessible fluid holds significance for various diseases and conditions. Further exploration of salivary biomarkers could lead to advancements in patient care, disease management, early detection, and potential cost reductions. This highlights the crucial role of salivary analysis in informing improved diagnostic and treatment strategies within dental practice.

## Acknowledgments

We would like to thank everyone who participated and made this research possible.

## Author contributions

**Conceptualization:** Aliaa Al Shaar, Omar Hamadeh, Ayman Ali.

**Data curation:** Aliaa Al Shaar.

**Investigation:** Aliaa Al Shaar, Omar Hamadeh, Ayman Ali.

**Methodology:** Aliaa Al Shaar, Omar Hamadeh, Ayman Ali.

**Project administration:** Omar Hamadeh, Ayman Ali.

**Resources:** Omar Hamadeh, Ayman Ali.

**Validation:** Aliaa Al Shaar, Omar Hamadeh, Ayman Ali.

**Visualization:** Aliaa Al Shaar.

**Writing – original draft:** Aliaa Al Shaar.

**Writing – review & editing:** Omar Hamadeh, Ayman Ali.
